# Comparison between Multi-Linear- and Radial-Basis-Function-Neural-Network-Based QSPR Models for The Prediction of The Critical Temperature, Critical Pressure and Acentric Factor of Organic Compounds

**DOI:** 10.3390/molecules23061379

**Published:** 2018-06-07

**Authors:** Mauro Banchero, Luigi Manna

**Affiliations:** Dipartimento Scienza Applicata e Tecnologia, Politecnico di Torino, Corso Duca degli Abruzzi, 24, 10129 Torino, Italy; luigi.manna@polito.it

**Keywords:** QSPR models, heuristic method, radial basis function neural networks, critical properties, acentric factor, molecular descriptors

## Abstract

Critical properties and acentric factor are widely used in phase equilibrium calculations but are difficult to evaluate with high accuracy for many organic compounds. Quantitative Structure-Property Relationship (QSPR) models are a powerful tool to establish accurate correlation between molecular properties and chemical structure. QSPR multi-linear (MLR) and radial basis-function-neural-network (RBFNN) models have been developed to predict the critical temperature, critical pressure and acentric factor of a database of 306 organic compounds. RBFNN models provided better data correlation and higher predictive capability (an AAD% of 0.92–2.0% for training and 1.7–4.8% for validation sets) than MLR models (an AAD% of 3.2–8.7% for training and 6.2–12.2% for validation sets). The RMSE of the RBFNN models was 20–30% of the MLR ones. The correlation and predictive performances of the models for critical temperature were higher than those for critical pressure and acentric factor, which was the most difficult property to predict. However, the RBFNN model for the acentric factor resulted in the lowest RMSE with respect to previous literature. The close relationship between the three properties resulted from the selected molecular descriptors, which are mostly related to molecular electronic charge distribution or polar interactions between molecules. QSPR correlations were compared with the most frequently used group-contribution methods over the same database of compounds: although the MLR models provided comparable results, the RBFNN ones resulted in significantly higher performance.

## 1. Introduction

The critical properties, such as the critical temperature (*T_c_*) and pressure (*P_c_*), of organic compounds are widely used in the chemical industry to understand the thermodynamic behavior of pure compounds or their mixtures, in particular when this is predicted through an equation of state. The acentric factor (*ω*) is also used in phase equilibrium calculations. It was first introduced by Pitzer to characterize the non-sphericity of molecular interactions, according to the following definition:

(1) $$\omega =-1-\log_{10}\left(p_{r}^{sat}\right){/}_{T_{r}=0.7}$$

Evaluating the acentric factor through its definition is often impossible for many compounds because the critical properties and/or the vapor pressure are experimentally unknown. Therefore, it is clear that estimation methods of both the acentric factor and the critical properties are necessary. Furthermore, high accuracy in estimation is required because phase equilibrium calculations are rather sensitive to these values [1].

“Group contribution methods” (GC methods) are the most commonly used estimation approaches. Typical GC methods for the critical properties and acentric factor are those proposed by Joback and Reid [2], Han and Peng [3] and by Gani’s research group [4,5,6]. The main advantage of these methods is that they are simple to use, but they also suffer from some disadvantages since not all groups are listed, the originally assumed additivity of the group contributions is often invalid and these methods are not always adequately accurate.

Quantitative Structure-Property Relationship (QSPR) models can also be used to perform property estimation since they establish quantitative correlations between diverse molecular properties and the chemical structure [7]. A QSPR model consists of a mathematical relationship between the property of interest and a variety of molecular features (named descriptors) derived from the structure of the molecule, which range from structural and topological indices to electronic and quanto-chemical properties.

The main steps of the method include database selection, molecular modelling of each chemical structure, molecular descriptor generation and selection, development of the mathematical relationship between the property and the selected descriptors as well as model validation. As far as the mathematical model is concerned, multi-linear regression (MLR) is the simplest and most commonly used approach in QSPR since it assumes a simple linear relation between the property and each molecular descriptor. However, nonlinear approaches, such as artificial neural networks (ANN), can also be employed. These approaches are able to “catch” hidden nonlinearities between the property and the descriptors, which make them better predictors than the MLR models in most cases [7]. 

The ANN “architecture” consists of a number of “neurons” that receive data from the outside, process the data using transformation functions and produce a signal. The “neurons” actually act as nonlinear transformation functions [7]. Many different ANN models and architectures can be found in the literature [8], among them, the Radial Basis Function Neural Networks (RBFNNs) may be easier to implement and offer shorter training times than standard feed-forward networks, even though they may require more neurons [9]. 

Previous literature reported QSPR MLR models to predict the critical temperature [10,11,12,13,14,15] and pressure [12,13,14,15] of organic compounds. Turner and coworkers [12], for example, developed eight-descriptor-MLR correlations over a database of 165 compounds, which mainly included hydrocarbons and oxygen-containing compounds, and obtained good results for both the critical temperature and pressure. Moreover, they pointed out that the prediction of compounds with high critical pressures produced most of the errors in their model. The same database was employed by Duchowicz and Castro [13] to predict critical properties by means of simple topological descriptors derived from atoms and classical bonds. In a previous study [14] a database of 132 diverse compounds was employed to predict the critical properties, and the obtained MLR models showed a significantly higher accuracy than GC methods as well as results that were comparable with other QSPR models, despite the different composition of the database. Recently, Sobati and Abooali [15] used an enhanced replaced method to develop simple and accurate QSPR MLR models to estimate the critical properties of pure refrigerants.

The QSPR ANN models were also used to predict the critical temperatures and pressures. The first examples of ANN models were based on electrotopological descriptors and provided better extrapolation and predictive capabilities than GC methods [16,17]. Godavarthy and coworkers [18] developed QSPR models for critical temperatures and pressures over a database containing 73 classes of hydrocarbons and comparing linear and non-linear approaches, including ANN models and genetic algorithms. They pointed out that the resultant nonlinear QSPR models were capable of making excellent predictions compared to the linear ones. Gharagheizi and Mehrpooya [19] developed feed-forward QSPR ANN models to predict the critical properties of the sulfuric compounds present in petroleum cuts. Later, the same research group [20] developed an ANN model combined with a GC approach, in which the neurons of the feed-forward ANN did not receive molecular descriptors as input, but pre-defined functional groups proposed by the authors. As far as RBFNNs are concerned, only Yao and coworkers [21] developed a RBNF model to predict critical temperatures, and they found that the correlation performance was higher than that of an MLR model employing the same ten molecular descriptors as the input. A similar RBNF approach, which was limited to substituted benzenes, was developed by the same research group to predict critical pressures [22]. 

Despite that recent works that make use of other QSPR approaches to predict the critical properties of organic compounds, such as support vector regression [23] or nonlinear random forest learning algorithms [24], have also included the estimation of the acentric factor, the number of papers that can be found on MLR and ANN models for the prediction of this last property is quite limited. Many examples of ANN models involve the estimation of the acentric factor for petroleum fractions. These models require other physical properties, such as the refractive index, the normal boiling point and the specific gravity or the molecular weight as input parameters [25,26,27]. Other examples are the above cited studies by Gharagheizi and coworkers, who also developed their sulfuric-compound [19] and GC-ANN [20] models for the acentric factor. 

In this work, the prediction performance of MLR models has been compared with that of RBFNNs, which were developed with the same database and the same molecular descriptors as the MLR models, in order to estimate the critical temperature, critical pressure and acentric factor. While examples of RBFNNs for the estimation of the critical temperature and pressure have already been reported in the literature [21,22], to the best of the authors’ knowledge, no examples can be found on the use of this ANN architecture for the prediction of the acentric factor. A database of 306 compounds, which included a large variety of structures (hydrocarbons, oxygenated, halogenated and nitrogenated organic compounds) was selected from the DIPPR database [28]. The models were then compared with the results reported in the literature for other QSPR approaches and with the GC methods proposed by Gani’s research group [4,5,6]. The comparison with the GC methods is particularly significant because the properties were calculated according to Gani’s procedure on the same database as the QSPR models of this work and because these methods are still among the most frequently used GC approaches to estimate the critical properties and acentric factors in phase equilibrium calculation. 

## 2. Results 

**Figure 1:**
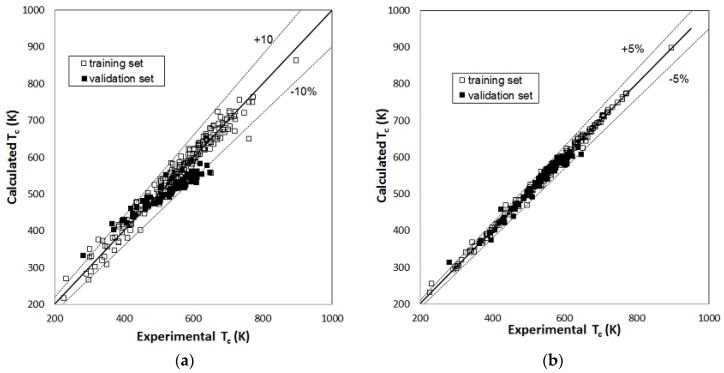
Parity plot of the calculated versus the experimental values of critical temperature: (**a**) MLR model; (**b**) RBFNN model.

**Figure 2:**
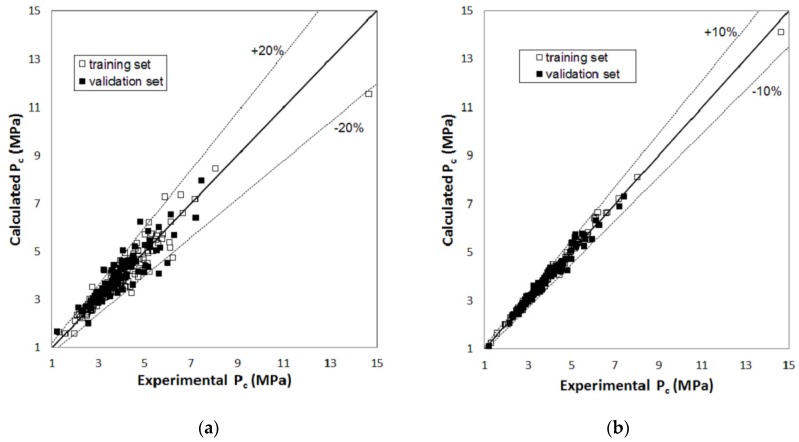
Parity plot of the calculated versus the experimental values of critical pressure: (**a**) MLR model; (**b**) RBFNN model.

**Figure 3:**
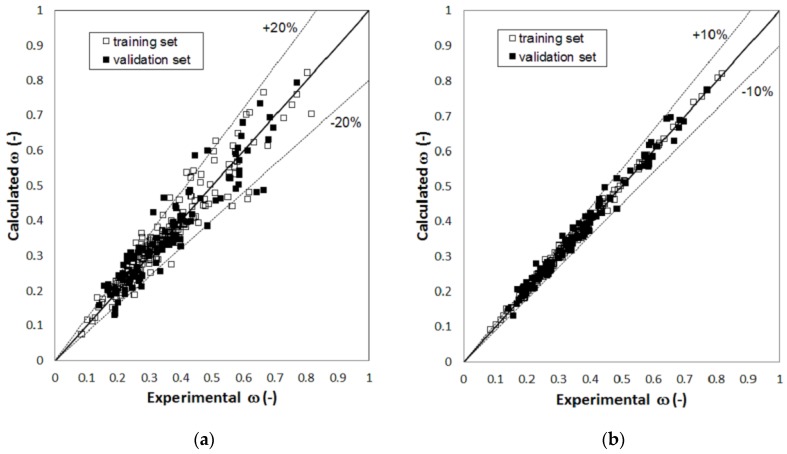
Parity plot of the calculated versus the experimental values of acentric factor: (**a**) MLR model; (**b**) RBFNN model.

Figure 1, Figure 2 and Figure 3 report parity plots that provide an indication of the accuracy of the correlations and a comparison of the MLR and RBFNN approaches. Table 1 reports a more detailed comparison between the results obtained for the training and the validation sets for the two models, in terms of statistical parameters, such as the average absolute percent deviation (AAD%) and the root mean square error (RMSE). It is clear from the data reported in Figure 1, Figure 2 and Figure 3 and Table 1 that the RBFNN models provide much better correlations of the data and better prediction abilities, with AAD% values ranging from 0.92 to 2.0% for the training sets and from 1.7 to 4.8% for the validation ones, compared to the MLR models. In fact, AAD% values for the latter range from 3.2 to 8.7% for the training sets and from 6.2 to 12.2% for the validation ones. Similarly, the RMSE values obtained with the RBFNN models are approximately equal to 20–30% of those obtained with the MLR models. The data in Table 1 also point out that the correlation performance and the predictive capability are higher for the models used for the critical temperature than those related to the critical pressure and acentric factor, which exhibits the worst result. In fact, it has already been pointed out in other studies [12,14] that the critical pressure is much more difficult to predict than the critical temperature. Furthermore, it is not surprising that the prediction of the acentric factor is even more problematic since it not only depends on the critical properties but also on the vapor pressure of each compound. The above order of the difficulty in prediction is also confirmed by the increasing number of the neurons in the hidden layer of the RBFNN models, which is equal to 42, 44 and 57 for critical temperature, critical pressure and acentric factor, respectively.

Table 2 reports a comparison of the correlation performance of the MLR and RBFNN models obtained in this work with previous QSPR models available in the literature [10,11,12,14,15,17,19,21,22]. The comparison is based on the RMSE, which is the most frequent statistical parameter provided by the different authors. Data in Table 2 clearly point out that the correlation performance of this work is comparable with those of previous studies, especially as far as the ANN models are concerned. Among the others, Yao and coworkers [21,22] were the only ones who used an RBFNN approach to evaluate critical temperatures and pressures, and their RMSE values are quite similar to those obtained in this work for the same properties. Table 2 also confirms that very few studies report prediction models for the acentric factor and points out that the RBFNN here proposed resulted in the lowest RMSE. However, in the authors’ opinion the comparison with previous literature is not very easy to discuss since the various models were developed with specific databases, which differ in terms of the number of compounds and the investigated chemical families. For this reason, the critical properties and acentric factor of the compounds selected in this work were calculated according to Gani’s GC methods [4,5,6] to perform a direct comparison with the same database. Gani’s approach was selected because it is the most accurate among the GC methods and because this class of estimation techniques is still widely used to estimate the critical properties and acentric factors in phase equilibrium calculation.

In Table 3, the performance of the QSPR correlations obtained in this work and that of Gani’s GC methods over the same database are compared. The GC methods confirm the same trend of the difficulty in prediction as the QSPR models, with the critical temperature displaying the lowest AAD% value and the acentric factor the highest. The MLR models provide a correlation performance that can be considered comparable with the GC methods, as far as the AAD% and the RMSE values are concerned. However, the RBFNN models provide significantly higher correlation performances with considerably lower values of the AAD% and RMSE values, which are reduced to 15–25% of those obtained with the GC methods.

## 3. Discussion

Molecular descriptors were selected by the heuristic algorithm to develop the MLR QSPR models for the critical temperature, critical pressure and acentric factor. The same descriptors were then used in the non-linear RBFNN models. 

The critical temperature has been found to depend on four constitutional, one geometrical, three electrostatic and two topological descriptors (Table 4). Constitutional descriptors are the simplest group of descriptors since they only reflect the molecular composition of the compound without using the geometry or electronic structure of the molecule. Among those reported in Table 4 for the critical temperature, one is related to the number of fluorine atoms while another two depend on the presence of the aromatic rings; the same or similar constitutional descriptors were also used in previous work to predict the critical temperature [18,19,20,21]. On the other hand, geometrical descriptors require 3D-coordinates of the atoms in the given molecule. In this work, the critical temperature has been found to depend on the moment of inertia B, which is the moment of inertia of the molecule with respect to the y-axis in the rigid rotor approximation [29].

The topographic electronic index (all pairs) is another descriptor that affects the critical temperature (Table 4). This is an electrostatic descriptor and it is calculated from knowledge of the partial atomic charges and the interatomic distances between all the pairs of atoms in the molecule. This type of descriptor reflects how differences in size, shape and constitution affect the electronic charge distribution and interatomic distances of the molecules [29]. The other two electrostatic descriptors reported in Table 4 for the critical temperature belong to the charged partial surface area (CPSA) class, which combines shape and electronic information to encode the features responsible for polar interactions between the molecules. Moreover, HASA_2_/TMSA^1/2^ and HDCA_2_/TMSA, where TMSA is the total molecular surface area, are related to the ability of a compound to form hydrogen bonds, with HASA_2_ being equal to the sum of the surface areas of all the H-bond acceptor atoms and HDCA_2_ being the solvent-accessible surface area of the hydrogen donor atoms [29]. These types of electrostatic descriptors and, in particular, the topographic electronic index (all pairs) and HDCA_2_/TMSA were also employed in previous studies to build-up QSPR correlations to predict the critical temperature of organic compounds [14,18].

The last two descriptors reported in Table 4 for the critical temperature are topological. Topological descriptors describe the atomic connectivity in the molecule that is characterized using graph invariants. These descriptors quantify various aspects of molecular architecture including shape, size, complexity and branching [30]. The Randic index (order 1) places emphasis on the bimolecular encounter that can occur among molecules and can be interpreted as the contribution of one molecule to the bimolecular interaction that arises from the encountering of the bonds of two identical molecules [29]. The structural information content (order 0) belongs to a class of topological indices that measures the complexity of the molecule in its graph representation [31]. These types of topological descriptors were also used in previous studies on the prediction of the critical temperature [14,18].

The critical pressure has been found to depend on two constitutional, two geometrical and six electrostatic descriptors (Table 4). One constitutional descriptor, the relative number of rings, is involved in both the correlations for the critical temperature and pressure, thus pointing out that the aromatic character of the molecule plays an important role in the prediction of both physical properties. As far as the geometrical descriptors are concerned, the rotational dynamics of the molecule was also found to affect the critical pressure although, unlike the critical temperature in which the moment of inertia B was involved, here, the moment of inertia C, which is calculated with respect to the z-axis, has to be used.

Electrostatic descriptors play a major role in the QSPR correlations for the critical pressure. This has already been pointed out in one of the authors’ previous work [14] in which five out of eight descriptors were electrostatic. The count of H-donors sites reported in Table 4 for the critical pressure is a simple electrostatic descriptor based on the counting of the number of H-donor sites in a molecule. The other descriptors for the critical pressure belong to the CPSA class, as already reported for critical temperature. HASA_1_ and HDSA_1_ are variants of the HASA_2_ and HDCA_2_ descriptors, respectively, which were found to influence the critical temperature, and to account for hydrogen bond interactions. FPSA_3_, which was also used in the correlations proposed by Godavarthy and coworkers [18], is equal to the atomic charge weighted positive surface area divided by the total molecular solvent-accessible surface area while the relative negative charged SA and the relative positive charged SA are equal to the solvent-accessible surface area of the most negative or the most positive atom, divided by the relative negative or positive charge, respectively [29]. The above results point out that polar interactions between molecules, with particular focus on hydrogen bonding, have a significant effect on the critical pressure. This may be due to the fact that secondary bonds between molecules become more significant as pressure is increased. 

The acentric factor has been found to depend on one constitutional, two geometrical, five electrostatic and two topological descriptors (Table 4). In analogy with the critical pressure, electrostatic descriptors also play a significant role in the acentric factor. Furthermore, one electrostatic descriptor, the count of H-donors sites, has also been used in the QSPR models of this present work for the critical pressure (Table 4). The other descriptors reported in Table 4 for the acentric factor are equal or very similar to those involved in the correlations of the critical temperature. The structural information content (order 0) and HDCA_2_, for example, were also used for the critical temperature even though the latter had to be divided by the TMSA. Moreover, the topographic electronic index required for the QSPR models of acentric factor refers to all bonded atoms in the molecule, while that required for the critical temperature refers to all pairs of atoms in the molecule. On the other hand, PNSA_3_, which is the sum of the solvent accessible surface areas of the atoms and the partial charges over all negatively charged atoms, belongs to the CPSA class [29] like many other electrostatic descriptors used to build the critical temperature and pressure correlations. Finally, the Kier & Hall index (order 2) is a topological connectivity index similar to the Randic index (order 1), which was employed for the critical temperature. The two indices share the same general formula but, while the Randic index requires the coordination numbers of each atom, the Kier & Hall index is a function of the atomic connectivity, which depends on the number of valence electrons, the atomic number and the number of hydrogen atoms connected to the i-th atom [30].

The above discussion has pointed out that six out of ten descriptors employed to develop the QSPR models for the acentric factor can be related to descriptors employed for the critical temperature and the critical pressure. This is not surprising since the acentric factor is closely dependent on these parameters. Furthermore, most of these descriptors are again connected to the electronic charge distribution in the molecule or to the secondary bonds between molecules, which is also confirmed by the other two of the last four descriptors reported in Table 4: the gravitation index (all bonds) reflects the mass distribution within the molecular space and quantifies the bulk cohesiveness of a compound due to the dispersion interactions [32], while the polarity parameter is the difference between the maximum and minimum partial charges in the molecule, which are calculated according to the approach proposed by Zefirov and coworkers [33].

## 4. Methods 

### 4.1. Database Selection

The data set consists of 306 organic compounds collected from the DIPPR database [28]. The compounds were selected after fixing a maximum relative error on the reported values of *T_c_* and *P_c_* to guarantee that the correlations were built over a database of compounds with comparable uncertainty. The uncertainty of the acentric factor is not reported in the DIPPR database, but it depends on the *T_c_* and *P_c_* present in the corresponding reduced coordinates. Only the compounds with a lower relative error than 1% in *T_c_* and 3% in *P_c_*, which are a great part of those reported in the database, were selected. *P_c_* measurements are generally affected by greater error than *T_c_* ones, thus a higher error was tolerated for the *P_c_* in order to have an adequate number of structures in the database. The complete list of the selected compounds is reported in Table S1 (supplementary material); the database includes as many chemical families as possible: linear and branched hydrocarbons (23.2%), cyclic (7.8%) and aromatic (8.2%) compounds as well as oxygenated (34.0%), halogenated (18.3%) and nitrogenated (8.5%) structures. The data set was randomly divided into two subsets: a training set of 215 compounds to build the correlations, and a validation set of 91 compounds to test the correlations. 

### 4.2. Molecular Modelling and Descriptor Generation

All the molecules were drawn using AMPAC [34] and were geometrically optimized using the semi-empirical AM1 model included in the software [35]. AM1 is an NDDO (Neglect of Diatomic Differential Overlap) semi-empirical model that only neglects the differential overlap for atomic orbitals on different atoms. AM1 calculations are fast and reasonably robust over a large range of chemical functionalities and AM1 continues to be used for a wide variety of applications [36]. Geometry optimization was carried out by searching for local minima on the potential energy surface, which is the hypersurface that represents the potential energy of the collection of atoms in the structure over all the possible atomic arrangements. The TRUSTE algorithm implemented in the AMPAC software was used to identify the lower energy configuration: the root mean square gradient tolerance was set equal to 0.05. The results were transferred to CODESSA, a software that can calculate constitutional, topological, geometrical, electrostatic, quantum-chemical and thermodynamic descriptors [37]. About 400 molecular descriptors were calculated for each compound. A pre-selection of the descriptors was performed, removing those not available for each structure and those with a constant value for all structures.

### 4.3. Multi-Linear Regression Correlations

In MLR models, the contribution of each descriptor is assumed to be linear, and a multi-parameter correlation with the following form is developed:

(2) $$Y=a_{0}+a_{1}X_{1}+a_{2}X_{2}+\ldots +a_{n}X_{n},$$

where *X_i_* (*i* = 1…*n*) is the ith descriptor, a*_i_* (*i* = 1…*n*) are the regression coefficients and *Y* is the property that has to be calculated.

The heuristic algorithm was chosen to develop the correlation: starting from one-parameter correlations, multi-linear ones are developed in a step-by-step procedure [37]. According to this method, one-parameter correlations are first considered and the descriptors with the Fisher *F*-test value or the R^2^ value or the Student *t*-value lower than 1.0, 0.1 or 1.5, respectively, are eliminated. If two descriptors are considered collinear, which happens if their pair-correlation coefficient exceeds a user-defined value (0.1), only the descriptor with the higher R^2^ is retained for further investigation. All the remaining descriptors are then listed in decreasing order, according to the one-parameter R^2^, and the selection of the best correlations proceeds starting from the top by adding non-collinear descriptors to each one-parameter correlation and checking for the fulfilment of the appropriate *t*-test and *F*-test conditions. The best two-parameter correlations (those with the highest *F*-value) are subjected to a similar procedure in order to add another descriptor. If the three-parameter correlation is found to be more significant than the two-parameter one, it is considered for further treatment. The procedure automatically adds one non-collinear descriptor at a time until the maximum number of descriptors (equal to ten as recommended by the default heuristic algorithm of CODESSA) has been reached. The selection of non-collinear descriptors, which were those with pair-correlation and significant pair-correlation coefficients lower than 0.99 and 0.8 [14], respectively, prevents chance correlations in the final QSPR models. The final results are the lists of the ten correlations with the highest R^2^ and the ten correlations with the highest *F*-values. The optimal correlation is defined as that with the highest R^2^ of the ten best according to the *F*-value. Further details of this procedure can be found in a previous study [14]. 

### 4.4. Radial Basis Function Neural Networks

An RBFNN generally has a feed-forward three-layer architecture with an input layer, a hidden radial basis layer and an output linear layer. The transfer function of the neurons in the hidden layer is a radial basis function that performs a nonlinear transformation of the input vector, $\bar{X}$, by calculating its Euclidean distance from the corresponding vector center, ${\bar{\mu }}_{j}$, as follows:

(3) $$f_{j}\left(\bar{X}\right)=\exp\left(-b_{j}^{2}\|\bar{X}-{\bar{\mu }}_{j}{\|}^{2}\right),$$

The RBFNN models were developed and optimized using the neural network toolbox in MATLAB [38]. The newrb function implemented in MATLAB, which iteratively creates a radial basis network by adding one neuron at a time, was used to adjust the network parameters. Neurons were added to the network until the mean squared error fell below a set error cut-off point (goal), which is subjected to an optimization procedure described at the end of the paragraph, or a maximum number of neurons, which was fixed at 30% of the number of compounds in the database, was reached. At each iteration, the input vector that lowers the network error the most was used to create a radial basis function neuron. The error of the new network was checked, and if it was low enough, the procedure was terminated, otherwise the next neuron was added. This procedure was repeated until the set goal or the maximum number of neurons was reached. To prevent overfitting of the training data, each network was used to calculate the mean squared error for the validation set (Section 4.5), and the whole procedure was repeated for different values of the goal until the error for the validation set was minimized. 

### 4.5. Model Validation

The accuracy of the correlations was tested by using them to predict the property of interest for an appropriate validation set. This set is composed of similar substances to those of the training set and for which the property of interest is experimentally known. The predictive capability of the correlation is evaluated by comparing the predicted and the experimental value of each property. To evaluate the quality of the correlation performance of the QSPR models, the critical properties and acentric factors of all substances in the database were also calculated according the Gani’s GC procedure reported in the literature [4,5,6]. Statistical parameters, such as the RMSE and the AAD%, were used to compare the correlation performance and the predictive capabilities of the MLR and the RBFNN models. 

## 5. Conclusions

QSPR MLR and RBFNN models have been developed to predict the critical temperature, critical pressure and acentric factor over a database of 306 organic compounds. To the best of the authors’ knowledge, this is the first time that the use of the RBFNN approach for the acentric factor has been reported. 

The two models, which were developed over the same molecular descriptors for each property, have been compared by means of statistical parameters, such as the AAD% and the RMSE. The results show that the RBFNN models provide much better correlations of the data and have a higher prediction capability to point out the non-linear nature of the relationship between these physical properties and the molecular structure. The results also show that the acentric factor is the most difficult property to predict since it depends on both the critical properties and also on the vapor pressure of each compound. 

The close relationship between the critical temperature, critical pressure and acentric factor has been pointed out by the nature of the selected molecular descriptors. Six out ten descriptors employed to develop the QSPR models for the acentric factor can be related to descriptors employed for the critical temperature and critical pressure. Even though the descriptors employed to build-up the correlations belong to different classes (i.e. constitutional, geometrical, topological and electrostatic classes), most of them are connected to the electronic charge distribution in the molecule or the polar interactions between molecules, especially as far as the critical pressure and acentric factor are concerned. This could suggest that the critical point of a substance is affected to a great extent by the secondary bonds between the molecules.

As far as the critical temperature and pressure are concerned, the QSPR methods obtained in this work resulted in similar correlation performances of those obtained in previous literature with different databases, while the lowest RMSE was found for the acentric factor. The quality of the QSPR correlations has also been compared with that of Gani’s GC methods over the same database. While the MLR models provided a comparable correlation performance with the GC methods, the RBFNN models resulted in significantly higher correlation performances, with considerably lower values of the AAD% and RMSE, which have been reduced by 15–25% of those obtained with the GC methods.

## Figures and Tables

**Table 1:** Comparison between the MLR and the RBFNN models for critical temperature, critical pressure and acentric factor.

		MLR Model		RBFNN Model	
		Training Set	Validation Set	Training Set	Validation Set
*T_c_*	total number of compounds	215	91	215	91
	compounds with AD% > 10%	8	9	-	1
	compounds with AD% < 5%	184	49	203	80
	AAD%	3.2%	6.2%	0.92%	1.7%
	RMSE (K)	22.0	37.4	7.2	11.9
*P_c_*	total number of compounds	215	91	215	91
	compounds with AD% > 10%	40	25	-	3
	compounds with AD% < 5%	124	45	171	60
	AAD%	6.1%	8.5%	1.9%	3.5%
	RMSE (MPa)	0.40	0.47	0.11	0.18
*ω*	total number of compounds	215	91	215	91
	compounds with AD% > 10%	65	45	1	7
	compounds with AD% < 5%	98	25	168	39
	AAD%	8.7%	12.2%	2.0%	4.8%
	RMSE (−)	0.040	0.066	0.0086	0.023

**Table 2:** Comparison between this work and literature QSPR methods for critical temperature, critical pressure and acentric factor.

		*T_c_*	*P_c_*	*ω*
RMSE for MLR models	Egolf and coworkers [10]	12 K	-	-
	Katritzky and coworkers [11]	15 K	-	-
	Turner and coworkers [12]	7.7 K	0.16 MPa	-
	Sola and coworkers [14]	12 K	0.25 MPa	-
	Sobati and Abooali [15]	16.3 K	0.27 MPa	-
	this work ^(1)^	27.5 K	0.42 MPa	0.049
RMSE for ANN models	Espinosa and coworkers [17]	5.6 K	0.08 MPa	-
	Gharagheizi and Mehrpooya [19]	18 K	0.17 MPa	0.032
	Yao and coworkers [21]	14 K	-	-
	Yao and coworkers [22]		0.15 MPa	-
	this work ^(1)^	8.8 K	0.13 MPa	0.015

^(1)^ Calculated on the whole database (training + validation sets).

**Table 3:** Comparison between MLR, RBFNN and Gani’s GC methods [4,5,6] for critical temperature, critical pressure and acentric factor.

		*T_c_*	*P_c_*	*ω*
AAD%	MLR model ^(1)^	4.1%	6.8%	9.7%
	RBFNN model ^(1)^	1.2%	2.3%	2.8%
	Gani’s GC method ^(2)^	2.7%	8.5%	14.1%
RMSE	MLR model ^(1)^	27.5 K	0.42 MPa	0.049
	RBFNN model ^(1)^	8.8 K	0.13 MPa	0.015
	Gani’s GC method ^(2)^	31.1 K	0.48 MPa	0.099

^(1)^ Calculated on the whole database (training + validation sets); ^(2)^ AAD% and *RMSE* for Gani’s GC methods were calculated on the same database of this work.

**Table 4:** Selected molecular descriptors for the QSPR models of critical temperature, critical pressure and acentric factor.

	Descriptor	Group
*T_c_*	Relative number of F atoms	Constitutional descriptor
	Number of aromatic bonds	Constitutional descriptor
	Relative number of rings	Constitutional descriptor
	Relative molecular weight	Constitutional descriptor
	Moment of inertia B	Geometrical descriptor
	HASA_2_/TMSA ^1/2^	Electrostatic descriptor
	HDCA_2_/TMSA	Electrostatic descriptor
	Topographic electronic index (all pairs)	Electrostatic descriptor
	Randic index (order 1)	Topological descriptor
	Structural Information content (order 0)	Topological descriptor
*P_c_*	Number of Cl atoms	Constitutional descriptor
	Relative number of rings	Constitutional descriptor
	Molecular volume	Geometrical descriptor
	Moment of inertia C	Geometrical descriptor
	HASA_1_	Electrostatic descriptor
	HDSA_1_/TMSA	Electrostatic descriptor
	FPSA_3_	Electrostatic descriptor
	Relative negative charged SA	Electrostatic descriptor
	Relative positive charged SA	Electrostatic descriptor
	count of H-donors sites	Electrostatic descriptor
*ω*	Relative number of double bonds	Constitutional descriptor
	Molecular surface area	Geometrical descriptor
	Gravitation index (all bonds)	Geometrical descriptor
	HDCA_2_	Electrostatic descriptor
	PNSA_3_	Electrostatic descriptor
	Polarity parameter (Q_max_ − Q_min_)	Electrostatic descriptor
	count of H-donors sites	Electrostatic descriptor
	Topographic electronic index (all bonds)	Electrostatic descriptor
	Structural Information content (order 0)	Topological descriptor
	Kier & Hall index (order 2)	Topological descriptor

## Supplementary Materials

The following are available online, Table S1: list of compounds collected from the DIPPR database and used to develop the QSPR models for critical temperature, critical pressure and acentric factor.

## Abbreviations

$\mathrm{AD}\%=\frac{\left|Y_{i}^{exp}-Y_{i}^{calc}\right|}{Y_{i}^{exp}}\times 100$	absolute percent relative deviation
$\mathrm{AAD}\%=\overset{n_{c}}{\underset{i=1}{\sum }}\frac{\left|Y_{i}^{exp}-Y_{i}^{calc}\right|}{Y_{i}^{exp}}\times \frac{100}{n_{c}}$	average absolute percent deviation
AM1	Austin Model 1
ANN	artificial neural network
GC	group contribution
MLR	multi-linear regression
NDDO	neglect of diatomic differential overlap
QSPR	quantitative structure-property relationship
RBFNN	radial basis function neural network
$\mathrm{RMSE}=\sqrt{\frac{\sum_{i=1}^{n_{c}}{\left(Y_{i}^{exp}-Y_{i}^{calc}\right)}^{2}}{n_{c}}}$	root mean square error
