# Supplementary material for: Comparison between Multi-Linear- and Radial-Basis-Function-Neural-Network-Based QSPR Models for The Prediction of The Critical Temperature, Critical Pressure and Acentric Factor of Organic Compounds
Source: Molecules. 2018 Jun 7;23(6):1379. doi: 10.3390/molecules23061379 (PMC6100065; doi:10.3390/molecules23061379)
Supplement: Supplementary file 1 [file molecules-23-01379-s001.pdf]

Table S1. List of compounds collected from the DIPPR database and used to develop the QSPR models for critical temperature, critical pressure and acentric factor.

	Compound name
1	1,1,1,2,3,3-hexafluoropropane
2	1,1,1,2-tetrafluoroethane
3	1,1,1-trichloroethane (3)
4	1,1,1-trifluoroethane (3)
5	1,1,2-trichlorotrifluoroethane
6	1,1-dichloro-1-fluoroethane (3)
7	1,1-dichloroethane (2)
8	1,1-difluoroethane (2)
9	1,1-difluoroethylene (2)
10	1,2,3-trimethylbenzene (2) (3)
11	1,2,4,5-tetramethylbenzene (3)
12	1,2,4-trimethylbenzene (3)
13	1,2-dibromotetrafluoroethane
14	1,2-dichlorotetrafluoroethane (3)
15	1,2-dimethoxyethane (2) (3)
16	1,3-butadiene (1)
17	1,4-dioxane (3)
18	1,6-hexane diamine
19	1-butanol (2) (3)
20	1-butene (1)
21	1-chloro-1,1-difluoroethane (2) (3)
22	1-decanol
23	1-dodecanol
24	1-heptanol
25	1-heptene (1)
26	1-hexanol (2) (3)
27	1-hexene (1)
28	1-methyl-2-pyrrolidinone
29	1-methylnaphthalene
30	1-nonanol (3)
31	1-nonene (1)
32	1-octanol (2)
33	1-octene (1)
34	1-pentanamine (3)
35	1-pentanol
36	1-pentene (1)
37	1-propanol
38	2-(2-butoxyethoxy) ethanol (3)
39	2,2,3,3-tetramethyilhexane (1)
40	2,2,3,3-tetramethylpentane (1)
41	2,2,3,4-tetramethylpentane (1)
42	2,2,3-trimethylbutane (1)
43	2,2,3-trimethylpentane (1)
44	2,2,4,4-tetramethylpentane (1)
45	2,2,4-trimethylpentane (1)
46	2,2,5,5-tetramethylhexane (1)
47	2,2-dichloro-1,1,1-trifluoroethane (3)
48	2,2-dimethylbutane (1)
49	2,2-dimethylheptane (1)
50	2,2-dimethylhexane (1)

51	2,2-dimethylpentane (1)
52	2,3,3,4-tetramethylpentane (1)
53	2,3,3-trimethylpentane (1)
54	2,3,4-trimethylpentane (1)
55	2,3-dimethylbutane (1)
56	2,3-dimethylhexane (1)
57	2,3-dimethylpentane (1)
58	2,4-dimethylhexane (1)
59	2,4-dimethyl-pentane (1)
60	2,5-dimethylhexane (1)
61	2-butanol (2) (3)
62	2-butoxyethanol (2)
63	2-chloro-1,1,1,2-tetrafluoroethane
64	2-ethoxyethilacetate (2)
65	2-heptanol (2)
66	2-heptanone
67	2-hexanol (2)
68	2-hexanone (3)
69	2-methyl 1-butene (1)
70	2-methyl 1-pentanol
71	2-methyl 3-ethylpentane (1)
72	2-methyl-1-butanol
73	2-methyl-1-propanol (2) (3)
74	2-methyl-2-butanol (2)
75	2-methyl-2-propanol (2)
76	2-methyleptane (1)
77	2-methylhexane (1)
78	2-methylnaphthalene (2) (3)
79	2-methyloctane (1)
80	2-methylpentane (1)
81	2-methylpyridine (2)
82	2-nonanol (3)
83	2-octanol (2) (3)
84	2-pentanol (2) (3)
85	2-pentanone (3)
86	2-propanol, 1,1′-iminobis (2) (3)
87	3,3,5-trimethylheptane (1)
88	3,3-dimethylhexane (1)
89	3,3-dimethylpentane (1)
90	3,4-dimethylhexane (1)
91	3-ethylhexane (1)
92	3-ethylpentane (1)
93	3-hexanone
94	3-methyl 1-butanol (3)
95	3-methyl 1-butene (1)
96	3-methyl 1-pentanol (2) (3)
97	3-methyl 2-butanol
98	3-methyl 3-ethylpentane (1)
99	3-methyleptane (1)
100	3-methylhexane (1)
101	3-methylpentane (1)
102	3-pentanone (3)
103	4-methyleptane (1)
104	5-nonanone (3)
105	acetal (3)
106	acetamide

107 acetic acid
108 acetone
109 acetonitrile (2) (3)
110 acetophenone (2)
111 anisole
112 benzenamine,3-methyl-
113 benzene
114 benzene, penthyl- (2)
115 benzene,1-ethyl-3methyl-
116 biphenyl (3)
117 bromobenzene (3)
118 bromoethane
119 bromotrifluoromethane (2) (3)
120 butanal
121 carbon tetrafluoride (3)
122 chlorodifluoromethane (3)
123 chloropentafluoroethane
124 chlorotrifluoromethane (2) (3)
125 chlorotrimethylsilane (2) (3)
126 cis 2-butene (1)
127 cis 2-pentene (1)
128 cumene (3)
129 cyclobutane (1)
130 cycloexane,1,1-dimethyl- (1)
131 cycloexane,1,3-dimethyl-,cis- (1)
132 cycloexane,1,3-dimethyl-,trans (1)
133 cycloexane,1,4-dimethyl-,cis- (1)
134 cycloexane,ethyl- (1)
135 cycloexane,propyl- (1)
136 cycloexene,1,2-dimetyl-,cis- (1)
137 cycloexene,1,2-dimetyl-,trans- (1)
138 cycloheptane (1)
139 cyclohexane (1)
140 cyclohexanol
141 cyclooctane (1)
142 cyclopentane (1)
143 cyclopentane,1,1-dimethyl- (1)
144 cyclopentane,1,2-dimethyl-,cis- (1)
145 cyclopentane,1,2-dimethyl-,trans (1)
146 cyclopentane,1,3-dimethyl-,cis- (1)
147 cyclopentane,1,3-dimethyl-,trans- (1)
148 cyclopentene (1)
149 cyclopropane (1)
150 decafluorobutane (2)
151 decanal (2)
152 dibenzothiophene (2)
153 dichlorodifluoromethane (2)
154 dichlorodimethylsilane (2) (3)
155 dichlorofluoromethane
156 dichloromethane
157 diethylamine (2)
158 diethylene glycol monopropylether (2)
159 diethylether (2)
160 difluoromethane (3)
161 diisopropylamine (2) (3)
162 diisopropylether (2)

163	dimethylether
164	di-n-butylamine (3)
165	di-n-octylamine (2)
166	di-n-propylether
167	ethane,2-(difluoromethoxy)-1,1,1-trifluoro-(3)
168	ethanethioic acid, s-ethyl ester (2)
169	ethanol (3)
170	ethene, 2-chloro-1,1-difluoroethene (2)
171	ether, bis(difloromethyl) (2)
172	ethyl acetate (2) (3)
173	ethyl chloride (2)
174	ethyl fluoride (2)
175	ethyl formate
176	ethyl propionate
177	ethyl-3-ethoxypropionate
178	ethylamine
179	ethylbenzene (3)
180	ethylcyclopentane (1)
181	ethylene (1)
182	ethylene glycol monopropyl ether (2) (3)
183	ethyne (2) (3)
184	fluorobenzene
185	formic acid,propilester
186	furan (3)
187	heptanal (2) (3)
188	hexafluorobenzene (2)
189	hexafluoroethane
190	hexanal (2) (3)
191	hexane,2,2,5-trimethyl- (1)
192	hydrazine (3)
193	indane
194	iodobenzene (2) (3)
195	isobutane (1)
196	isobutylacetate (3)
197	isobutylbenzene
198	isobutyric acid (2) (3)
199	isopentane (1)
200	isopropanol (3)
201	isopropyl acetate (2)
202	isopropylamine
203	isovaleric acid (2) (3)
204	m-cresol (3)
205	mesitylene (2) (3)
206	methanamine,n-methyl-
207	methanethiol (2) (3)
208	methanol
209	methoxyacetone (2) (3)
210	methyl acetate (2)
211	methyl chloride
212	methyl ethylether (3)
213	methyl formate (2) (3)
214	methyl isobutylketone (2)
215	methyl isopropylether
216	methyl isopropylketone (2)
217	methyl n-butylether (2) (3)
218	methyl n-butyrate

219 methyl n-pentylether
220 methyl n-propylether
221 methyl propionate [(3)]
222 methyl tert-butylether [(2)] [(3)]
223 methylacetylene [(2)]
224 methylamine [(2)]
225 methylciclopentane [(1)]
226 methylcyclohexane [(1)]
227 methylethylketone [(2)]
228 methylfluoride
229 methylisobutyrate
230 m-xylene
231 n,n-dimethylaniline [(3)]
232 naphthalene
233 n-butane [(1)]
234 n-butylacetate [(3)]
235 n-butylamine [(2)]
236 n-butylbenzene [(2)]
237 n-butyric acid [(2)]
238 n-decane [(1)]
239 n-decanoic acid
240 neopentane [(1)]
241 n-heptane [(1)]
242 n-heptanoic acid [(2)]
243 n-hexane [(1)]
244 n-hexanoic acid [(2)]
245 nitrogen trifluoride
246 nitromethane [(2)] [(3)]
247 n-methylaniline [(2)]
248 n-nonane [(1)]
249 n-nonanoic acid [(3)]
250 n-octane [(1)]
251 n-octanoic acid
252 n-pentane [(1)]
253 n-pentanoic acid [(3)]
254 n-pentylacetate
255 n-propyl n-butyrate [(2)]
256 n-propyl propionate
257 n-propylacetate
258 n-propylamine
259 n-propylbenzene
260 n-propylchloride [(3)]
261 o-cresol [(2)]
262 octafluorocyclobutane
263 octafluoropropane [(3)]
264 octamethylcyclotetrasiloxane [(3)]
265 octanal
266 octane,3-methyl- [(1)]
267 octane,4-methyl- [(1)]
268 oxirane [(3)]
269 o-xylene [(2)]
270 p-cresol
271 p-cymene
272 p-diethylbenzene [(3)]
273 pentafluoroethane
274 pentanal [(2)]

275	pentane,3,3-diethyl- [(1)]
276	pentane,dodecafluoro-
277	perfluoro n-decane [(2)] [(3)]
278	perfluoromethylcyclopentane [(2)]
279	perfluoro-n-heptane
280	phenol
281	piperidine [(2)] [(3)]
282	propadiene [(1)]
283	propane [(1)]
284	propane,1,1,2,2,3-pentafluoro- [(3)]
285	propanoic acid,butylester [(2)] [(3)]
286	propionic acid
287	propionitrile [(3)]
288	propylene [(1)]
289	propylene glycol monomethyletheracetate[(2)]
290	p-xylene [(2)]
291	pyridine [(2)] [(3)]
292	pyrrolidine [(3)]
293	tetrachloromethane [(3)]
294	tetrafluoroethylene [(2)] [(3)]
295	tetrahydrofuran [(2)] [(3)]
296	thiobismethane [(2)] [(3)]
297	thiophene [(2)]
298	toluene [(3)]
299	trans 2-pentene [(1)]
300	trans-1,4-dimethylcyclohexane [(1)]
301	trichlorofluoromethane
302	triethylamine
303	trifluoroiodomethane
304	trifluoromethane
305	trimethylamine [(2)] [(3)]
306	vinyl fluoride [(2)] [(3)]

[(1)] compound in the validation set of the models for the critical temperature

[(2)] compound in the validation set of the models for the critical pressure

[(3)] compound in the validation set of the models for the acentric factor
